# COVID-19 and stroke MR study: data errors, timing gaps, statistical flaws

**DOI:** 10.1186/s12985-025-02852-1

**Published:** 2025-06-30

**Authors:** Xiaoyang Zhu, Dan He

**Affiliations:** 1 Binzhou Medical University, Yantai, Shandong China; 2 Qingdao Traditional Chinese Medicine Hospital, Qingdao Hiser Hospital Affiliated of Qingdao University, Qingdao, Shandong China

## Abstract

This correspondence critiques the Mendelian randomization study by Zhang et al. on the causal link between COVID-19 and ischemic stroke, highlighting three methodological concerns. First, the study incorrectly attributes the eQTL data to the GTEx database, whereas the actual source is the eQTLGen Consortium, necessitating correction for accurate data provenance. Second, the outcome data (collected prior to 2018) predate the COVID-19 pandemic (post-2019), violating the relevance assumption in instrumental variable analysis. This temporal mismatch may render observed associations biologically implausible, reflecting genetic pleiotropy or residual confounding rather than causal effects of SARS-CoV-2 infection. Third, the absence of multiple testing correction elevates the risk of false positives, undermining the validity of subsequent gene pathway analyses. Addressing these issues—revising data attribution, clarifying temporal limitations, and enhancing statistical rigor—would strengthen the study’s reliability and translational implications.

Dear Editor,

The study by Zhang et al., titled “*The causal association between COVID-19 and ischemic stroke: a mendelian randomization study*” [1], offers a compelling application of Mendelian randomization to unravel the genetic interplay between COVID-19 severity and ischemic stroke. The authors’ identification of ten shared putatively causal genes, including CFL2 and FAM20C, provides a Mendelian randomization framework for understanding thromboinflammatory mechanisms in COVID-19-related ischemic stroke. This work stands out for its rigorous design and potential clinical relevance, particularly in guiding targeted therapies for high-risk patients. However, to maximize the impact of these findings, several methodological and translational aspects merit elaboration.

The original text contains an inaccurate citation regarding data sources. Specifically, the manuscript erroneously attributes the acquired eQTL data to the GTEx database through the IEU Open GWAS platform (https://gwas.mrcieu.ac.uk/) [2]. However, this attribution is factually inconsistent because the IEU Open GWAS repository does not host GTEx datasets. Through careful examination of the eQTL data identifiers provided in the supplementary materials, we have identified that the referenced data actually originated from the eQTLGen Consortium, not from GTEx as originally claimed. This discrepancy requires immediate correction to ensure proper scientific documentation and data provenance [3]. This also implies that the eQTL data represented only blood tissue, not multiple tissues as claimed, and may mislead readers regarding the biological significance of the reported findings.

Secondly, the COVID-19 pandemic first emerged in late 2019, while the outcome data of gene eQTLs used in this study were exclusively measured prior to 2018. It is crucial to clarify that although the eQTLGen consortium’s original article was published in 2023 [3], the integrated datasets were collected before 2018, with the IEU Open GWAS project explicitly designating 2018 as the measurement date for this data. Since the genotyping of the outcome data from the eQTLGen Consortium was completed prior to the COVID-19 pandemic, the study population remained unexposed to SARS-CoV-2 infection. This temporal discrepancy creates a violation of the relevance assumption in instrumental variable analysis, as the genetic instruments lack actual association with the exposure (COVID-19) in the outcome sample. Consequently, the observed associations may primarily reflect underlying genetic pleiotropy or residual confounding rather than causal effects of COVID-19 infection, rendering the results biologically implausible as direct disease outcomes of viral infection [4].

Thirdly, the original study did not apply false discovery rate correction when identifying putatively trait-associated genes using Mendelian randomization analysis. This lack of multiple testing adjustment increases the risk of false positives and undermines the statistical credibility of the reported gene-trait associations. It also represents an additional methodological limitation of the study.

In summary, the study raises important hypotheses regarding potential genetic mechanisms linking COVID-19 and ischemic stroke, which require further validation. Addressing three critical issues - data attribution discrepancies, temporal validity concerns, and enhanced statistical rigor - would substantially strengthen the evidentiary basis and translational impact of these findings. We look forward to the authors’ forthcoming rigorous studies driving significant progress in the evolving domain of COVID research.

## Data Availability

No datasets were generated or analysed during the current study.
